# Recommendations for Standardizing Nuclear Medicine Terminology and Data in the Era of Theranostics and Artificial Intelligence

**DOI:** 10.2967/jnumed.124.269424

**Published:** 2025-09

**Authors:** Tyler J. Bradshaw, Julia Brosch-Lenz, Carlos Uribe, Nicolas Karakatsanis, Richard Bruce, Lidia Strigari, Abhinav Jha, Joyita Dutta, Jazmin Schwartz, Georges El Fakhri, Atlas Avval, Arman Rahmim, Babak Saboury

**Affiliations:** 1 Department of Radiology, University of Wisconsin–Madison, Madison, Wisconsin;; 2 Institute of Nuclear Medicine, Bethesda, Maryland;; 3 Molecular Imaging and Therapy, BC Cancer, Vancouver, British Columbia, Canada;; 4 Department of Integrative Oncology, BC Cancer Research Institute, Vancouver, British Columbia, Canada;; 5 Department of Radiology, University of British Columbia, Vancouver, British Columbia, Canada;; 6 Department of Radiology, Weill Cornell Medicine, New York, New York;; 7 Department of Medical Physics, Azienda Ospedaliero-Universitaria di Bologna, Bologna, Italy;; 8 Department of Biomedical Engineering and Mallinckrodt Institute of Radiology, Washington University in St. Louis, St. Louis, Missouri;; 9 Department of Biomedical Engineering, University of Massachusetts Amherst, Amherst, Massachusetts;; 10 Department of Medical Physics, Memorial Sloan Kettering Cancer Center, New York, New York;; 11 Departments of Radiology and Biomedical Engineering and Bioinformatics and Data Science, Yale University, New Haven, Connecticut;; 12 Mashhad University of Medical Sciences, Mashhad, Iran;; 13 Department of Radiology, Hospital of the University of Pennsylvania, Philadelphia, Pennsylvania; and; 14 United Theranostics, Bethesda, Maryland

**Keywords:** image processing, terminology standardization, artificial intelligence, data standardization, theranostics

## Abstract

There is a pressing need for improved standardization of terminology and data in nuclear medicine. The field is experiencing unprecedented growth, driven by advances in radiopharmaceutical therapy (RPT) and the emergence of artificial intelligence (AI). However, there are challenges that threaten to frustrate this continued progress. For instance, despite the successes of RPT, high-quality evidence on how to best personalize RPT and take full advantage of its theranostics properties is still lacking. To obtain this evidence, large, structured datasets are needed to associate different RPT strategies with patient outcomes. Large datasets are also needed for the development of AI algorithms, especially as new foundation models demand increasingly large training datasets. Both of these obstacles could be overcome by multiinstitutional data sharing. However, inconsistencies in terminology and data collection make effective data pooling difficult. This article, produced by the Society of Nuclear Medicine and Molecular Imaging AI–Dosimetry Working Group, discusses the need for standardization in nuclear medicine terminology and data. We advocate for the adoption of standardized data and metadata frameworks based on controlled biomedical ontologies to better harmonize the collection of nuclear medicine data. We provide recommendations for the field that, if followed, would facilitate multiinstitutional data sharing and allow for the collection of large datasets. We describe a use case demonstrating how standardized vocabularies and data collection can enhance efforts to associate theranostics target expression data with patient outcomes.

Nuclear medicine is a multidisciplinary field with a rich scientific lineage, drawing contributions from nuclear physics, radiation biology, radiology, imaging science, medical oncology, radiation oncology, pharmacology, radiochemistry, and others. This interdisciplinary synergy has fueled the recent growth of theranostics, culminating in successful phase 3 clinical trials of radiopharmaceutical therapies (RPTs) (1,2) that target the somatostatin receptors of neuroendocrine tumors and prostate-specific membrane antigen (PSMA) in prostate cancer (3,4). These achievements have ignited growing interest in the field. Consequently, an influx of investments from industry and government has quickly expanded the nuclear medicine research community, accelerating the rate of innovation (5,6).

However, this rapid expansion presents some unique challenges that will require multiinstitutional data sharing to overcome. Specifically, efforts to optimize RPT approaches and develop robust artificial intelligence (AI) models have been hindered by the lack of large, standardized nuclear medicine datasets. For example, the potential for dosimetry-guided RPT to improve outcomes is significant, but there is a lack of high-quality evidence showing when and how to use dosimetry to optimize treatments (7,8). Similarly, the trend toward large AI foundation models requires increasingly large training datasets, which is challenging for the nuclear medicine field because of its comparatively lower examination volumes.

These issues can be overcome by pooling datasets across institutions. However, inconsistencies in nuclear medicine terminology and data make this difficult. Inconsistencies in terminology can inhibit mutual understanding of concepts, making it difficult to combine, compare, and reuse data (9). Such inconsistencies can also hinder attempts to reproduce or build on research studies (10). The need for standardization also extends to clinical protocols and documentation, radiopharmaceutical production, equipment calibration, dosimetry processes, and others.

In this article, we explain the need for standardization of terminology and data collection in nuclear medicine. We provide a set of key steps that need to be addressed by the field to make it possible to efficiently and accurately pool data from multiple sources. These recommendations stem from the collective expertise of the Society of Nuclear Medicine and Molecular Imaging AI–Dosimetry Working Group and mirror standardization efforts that have occurred in other fields (11–15). To illustrate the importance of these principles, we examine an example research problem—correlating clinical outcomes with absorbed doses (ADs) from dosimetry studies in patients receiving PSMA RPT—and discuss how standardization can help address this problem.

## STANDARDIZING TERMINOLOGY

In any knowledge domain, it is important for terms and definitions to be agreed upon. At a basic level, standardized terminology leads to conceptual clarity, meaning that those within the field share a common understanding of concepts. This has many downstream benefits. It facilitates the standardization of practices and protocols, supports the exchange of data and information, optimizes data usability, and leads to more coherent knowledge integration so that researchers can build on each other’s research (16,17). This is especially critical in the emerging era of AI and large language models, where imaging and text datasets from different institutions must be combined and their metadata automatically scraped from electronic medical records (EMRs) to support algorithm development and testing (18,19).

### History of Medical Coding

Efforts to standardize concepts and nomenclature in medicine have been ongoing for over a century. One of the earliest and most significant developments was the *International Classification of Diseases* (20). This aimed to enable international comparability in health data by providing codes to classify diseases, signs, and symptoms. Another crucial development came in 1966 with the introduction of the *Current Procedural Terminology* by the American Medical Association (20), which serves as a comprehensive, precoordinated coding scheme for diagnostic and therapeutic procedures and interventions for the U.S. health care system. These medical coding initiatives have been pivotal in addressing inconsistencies and ambiguities in medical recordkeeping, billing, research, and communication.

### Biomedical Ontologies

**FIGURE 1.**
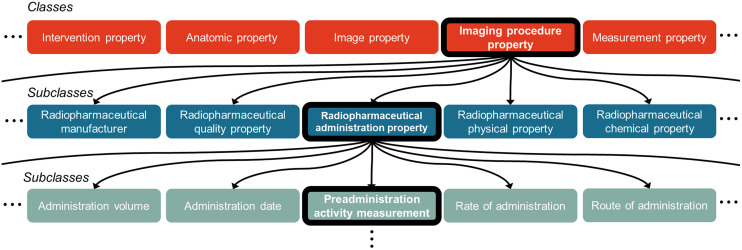
Example structure of hypothetical biomedical ontology for nuclear medicine concepts. Ontologies organize knowledge hierarchically using classes (general concepts, top row) and subclasses (more specific concepts, middle and bottom rows). Lines indicate an “is a” relationship, where a subclass is a type of its parent class. For instance, (the bolded path) “Preadministration Activity Measurement” is a type of “Radiopharmaceutical Administration Property,” which is a type of “Imaging Procedure Property.”

It became evident that efforts for terminology standardization needed to extend beyond medical coding to encompass a broader range of medical concepts (9). Ontologies became a powerful tool for accomplishing this. An ontology is a formal, structured list of concepts within a domain, describing key attributes and the relationships between them (16). They often consist of classes, which represent general concepts, and subclasses, which are more specific concepts within a category, organized in an “is a” hierarchical structure (e.g., PET scanner “is a” type of nuclear medicine scanner). They can also include definitions, synonyms, and preferred terms. A hypothetical ontology is illustrated in Figure 1.

Several biomedical ontologies play a crucial role in the U.S. health care system. One prominent example is the Systematized Nomenclature of Medicine Clinical Terms (SNOMED-CT), an ontology encompassing a vast array of biomedical concepts (21). Its predecessor, SNOMED, evolved from a pathology-specific nomenclature system. Another example is Logical Observation Identifiers Names and Codes (LOINC), which provides standardized terminology and coding for laboratory tests, health measurements, and clinical observations (22). Additionally, specialized ontologies have emerged in different medical domains. RadLex, an ontology of radiology terms for standardized indexing and retrieval of radiology information, was introduced in 2005 by the Radiological Society of North America (23). The oncology-focused National Cancer Institute Thesaurus is another widely adopted resource (24). Many of these ontologies are integrated into the Unified Medical Language System and are accessible through BioPortal, a Web-accessible repository for biomedical ontologies (25).

Despite these resources, terminology in nuclear medicine needs more comprehensive standardization to reflect its unique knowledge domain. The ontologies described above do not adequately cover key nuclear medicine concepts. For example, *theranostics*, *S value*, and *energy window* are not found in existing ontologies. Many terms are used interchangeably and without clear definitions or distinctions, such as *radiopharmaceutical therapy*, *molecular radiotherapy*, *internal radiation therapy*, *radioligand therapy*, and many more. Nuclear medicine concepts that are found in existing ontologies are often scattered throughout different ontologies with inconsistent groupings, making it difficult to draw relationships among them. This level of disorganization may be an obstacle to the growth and maturation of the field. Some prior efforts have proposed definitions for limited sets of nuclear medicine terms, such as the International Organization for Standardization (26) and *International Commission on Radiation Units and Measurements Report No. 96* (27), but a more comprehensive effort is needed to address any ambiguities across modern nuclear medicine terminology.

### Recommendations

To address the heterogeneity of terminology in nuclear medicine, we recommend 2 solutions. First, academic journals, professional organizations, and software developers should adopt terminology standards that align with existing nonconflicting ontologies. For journals, this means that their editorial style and nomenclature guide should link with Unified Medical Language System ontologies, particularly as it pertains to “preferred terms.” Professional societies should also adopt similar terminology guides for official reports, guidelines, education, and general material. Nuclear medicine training programs should ensure that educational content is consistent with ontologies. It is critically important that software and hardware developers use ontology-grounded terms, as scanners are a primary source of nuclear medicine data and metadata.

**FIGURE 2.**
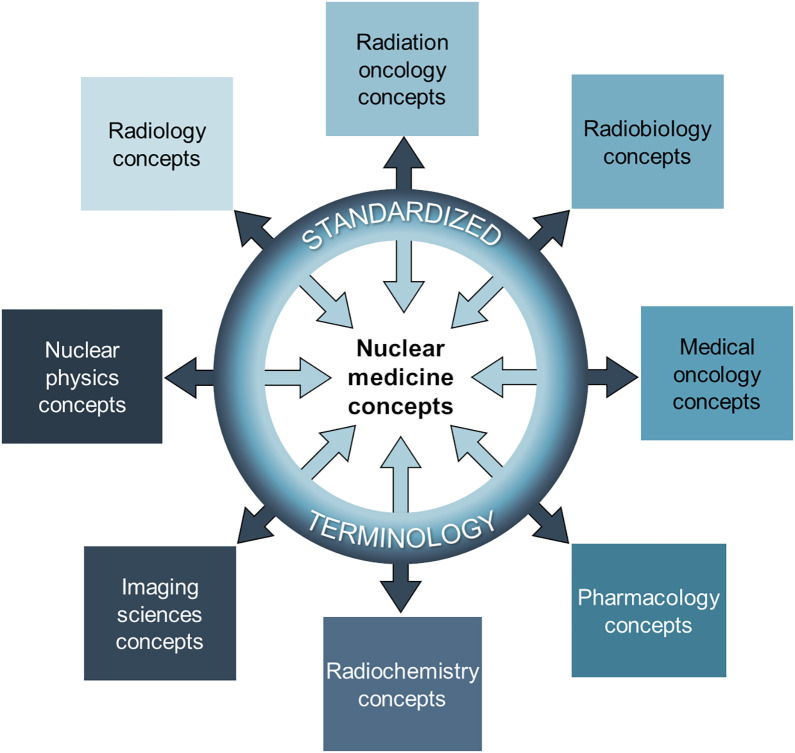
Role of standardized terminology in integrating diverse concepts within nuclear medicine. Nuclear medicine draws on numerous fields, each with its own unique concepts and vocabularies. Shared, standardized terminology is essential for ensuring that concepts from contributing disciplines are accurately understood, communicated, and integrated within the practice and study of nuclear medicine.

Second, there is a need for the nuclear medicine community to be more involved in the development of biomedical ontologies. This will help ensure that the unique terms and concepts in nuclear medicine are comprehensively covered, without conflicts, in biomedical ontologies (Fig. 2). This would involve systematically identifying nuclear medicine concepts that need to be accounted for, including all potential data elements that may be collected. One initiative currently underway is NucLex (28,29), which aims to improve existing ontologies, including RadLex, SNOMED-CT, and LOINC, so they better support concepts within nuclear medicine by clearing ambiguities and resolving conflicts among them. NucLex also aims to identify unsupported terms, provide definitions and synonyms, and link concepts across different ontologies, when possible. It will ultimately cover diagnostic and therapeutic nuclear medicine and research concepts (e.g., simulations, biologic modeling). This must be a perpetual, ongoing effort, as new concepts will continue to emerge. Another recent effort toward terminology standardization was the survey and subsequent report from the Nuclear Medicine Global Initiative on preferred terms for systemic radionuclide-based therapy (i.e., RPT), offering valuable insights into current terminology trends and heterogeneity (30).

### Use Case

To illustrate how ontologies can aid in specific use cases, we will examine the research problem of correlating AD measurements from RPT dosimetry with patient outcomes for PSMA RPT. By formally defining concepts and standardizing terminologies, ontologies can help ensure that comparisons and aggregations of AD measurements from different PSMA dosimetry studies are appropriate (31). This is essential because variations in dosimetry methods can lead to significant discrepancies in AD values, potentially confounding analyses. A key area where ontologies can offer conceptual clarity is in the disambiguation of metadata descriptors, such as imaging methods (e.g., planar vs. dual-head SPECT/CT vs. ring-shaped SPECT/CT), image processing (e.g., registration and segmentation methods), volume and mass calculation approaches, and dosimetry calculation methods.

For instance, in a study of a ^177^Lu dosimetry dataset that was shared among research teams, significant discrepancies in calculated AD values for kidneys, liver, spleen, and tumors emerged, highlighting the impact of differing dosimetry approaches (8). Notably, the use of planar images versus 3-dimensional SPECT/CT images led to systematic differences in AD values. Ontologies are needed so that these different concepts and methods are unambiguously defined and can easily be distinguished from one another. This is crucial for drawing valid conclusions and avoiding errors due to unaccounted-for systematic biases. Similarly, when AD values are reported for kidneys, it is critical to understand whether the measurement refers to both kidneys or the left or right kidney and which parts of the kidney (pelvis, medulla, cortex) were considered in the measurement. By providing precise definitions for these and other relevant concepts, a nuclear medicine ontology can enable accurate data description, interpretation, and valid comparison across studies.

## STANDARDIZING DATA

To effectively advance theranostics, researchers will need to combine datasets from multiple sources. To realize the full potential of multiinstitutional studies, data standards are needed. Data standardization is a critical early step in any effort to mitigate confounding effects and generate reliable evidence from observational data (32). Inconsistent or incompatible data formatting and labeling can lead to challenges in interoperability, data integration, reproducibility, interpretability, and comparative analysis (33).

**FIGURE 3.**
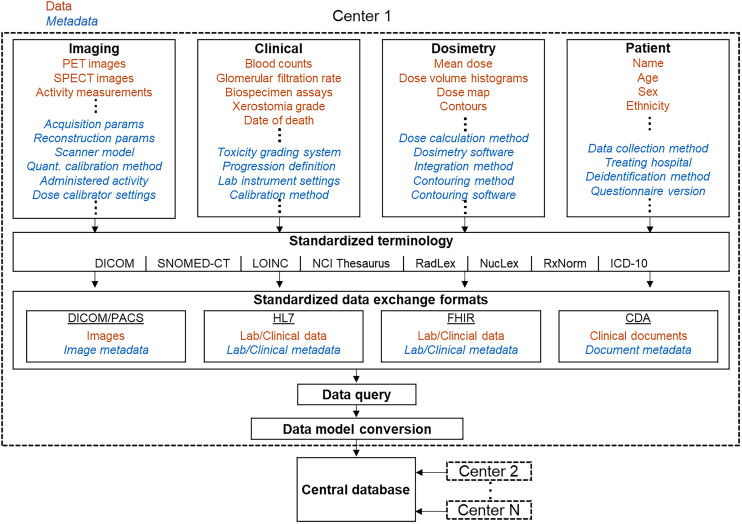
Conceptual workflow for aggregating multiinstitutional theranostics data. Diverse data (orange) and metadata (blue, italics) spanning Imaging, Clinical, Dosimetry, and Patient domains are collected at each center (dashed boxes). Applying standardized terminology (e.g., SNOMED-CT, LOINC, RadLex, NucLex) to data labels ensures consistent meaning between datasets. Standardized data exchange formats (e.g., DICOM, HL7, FHIR) facilitate interoperability. This structured data can then be queried from clinical databases, converted into a common format using a data model, and input into a central database, enabling pooling of information from multiple centers. CDA = clinical document architecture; *ICD-10* = *International Classification of Diseases, 10th Revision;* NCI = National Cancer Institute; PACS = picture archiving and communication system; Params = parameters; Quant. = quantitative.

Equal in importance to the standardization of data is the standardization of metadata. Metadata (i.e., “data about data”) give context to a dataset. Metadata can describe the source, creation method, structure, status, level, and semantics of a data object, and are the primary means by which data can be queried, organized, and reused (34). The Digital Imaging and Communications in Medicine (DICOM) standard, for instance, specifies the technical structure and requirements for capturing and transmitting data and metadata intrinsic to an imaging examination. One of the key features of the DICOM standard is that all medical imaging objects are self-described and contain sufficient information to fully describe the information about the patient, examination, and acquisition device and parameters. The metadata needed for theranostics, however, are much more comprehensive than the data required for imaging acquisition (Fig. 3). For instance, interpreting results from RPT dosimetry analysis requires knowledge of how the SPECT scanner was calibrated for quantitative imaging, how the organs and tumors were delineated, and the software and specific model parameters used for dosimetry calculations, among other factors. A minimum list of variables for reporting dosimetry results has been recently proposed (31), but a full list of relevant variables is likely much larger.

Data generation should be done in accordance with four guiding principles (findable, accessible, interoperable, and reusable), collectively known as FAIR. The FAIR principles, embraced by numerous funding agencies and research organizations, provide crucial guidelines for effective dataset curation, management, and sharing (10). To be compliant with FAIR principles, datasets must be collected and labeled with consideration to how they might be discovered and repurposed in the future. Ethical and legal issues should also be considered before data collection to ensure fairness, transparency, and trustworthiness (35–37).

Some efforts to centrally collect large multiinstitutional imaging datasets are underway, including The Cancer Imaging Archive (38) and the Medical Imaging and Data Resource Center (39). However, theranostics datasets are not well represented in these repositories. To address this gap, the Society of Nuclear Medicine and Molecular Imaging has initiated an effort to centrally collect theranostics datasets through the Radiopharmaceutical Therapy Registry (40). This registry involves multiple participating centers collecting theranostics imaging datasets and clinical information obtained through standardized questionnaire forms. However, expanded efforts to collect theranostics datasets that cover a diverse range of clinical data types and diseases are needed.

### Recommendations

We have several recommendations regarding the formatting, labeling, and generation of data to facilitate standardized data collection and sharing.

#### Data Model

To collect a standardized, FAIR, nuclear medicine dataset, a nuclear medicine data model is needed. A data model is a conceptual framework that defines how data are input, structured, and labeled within a database and serves as a blueprint for ensuring the consistency of data collected from multiple sources. A data model goes further than standards that address data-exchange formats and interchange protocols (e.g., DICOM, Health Level 7 [HL7] Fast Healthcare Interoperability Resources [FHIR]). A data model that is capable of capturing the multidisciplinary nature of nuclear medicine data, including imaging, therapy, dosimetry, laboratory test results, and clinical data, is needed.

One example of a data model that may serve the multidisciplinary needs of nuclear medicine is the open-source Observational Medical Outcomes Partnership (OMOP) Common Data Model (CDM), developed by Observational Health Data Sciences and Informatics (41). The OMOP CDM replicates a patient-centric medical record system, using standardized and structured vocabularies and a flexible design that can accommodate a range of longitudinal, observational health care data, and was recently expanded to handle imaging data measurements (42). Currently, the model may not be capable of fully capturing the nuances of nuclear medicine imaging and RPT datasets, such as multiple time point SPECT/CT imaging results with voxel-level dosimetry. However, a concerted effort by nuclear medicine experts collaborating with the Observational Health Data Sciences and Informatics community could lead to an enhanced CDM capable of handling these datasets.

Another potential data model is from the Clinical Data Interchange Standards Consortium (CDISC) (43). CDISC provides standards for standardizing, exchanging, and submitting clinical trial data. CDISC’s Study Data Tabulation Model can help organize, describe, and exchange data with traceability. It is also capable of modeling longitudinal data. The standards established by CDISC are required for data submission to regulatory bodies such as the Food and Drug Administration in the United States and the Pharmaceuticals and Medical Devices Agency in Japan. CDISC provides definitions and implementation guides for digital dataflow. Relations and dependencies within a clinical trial design are clearly mapped using a standardized vocabulary. Notably, the CDISC data model can be transformed into OMOP CDM (44).

Regardless of which data model framework is used, nuclear medicine experts need to make a concerted effort to generate a comprehensive list of data elements that may be collected as part of an imaging or therapy dataset. These data elements need to span different data domains, including imaging, dosimetry, laboratory, and clinical domains and therefore will likely require collaborations with experts from these different domains. The data elements must have accompanying metadata grounded in established ontologies so that data objects can be organized and queried. A data dictionary should provide detailed descriptions of each data element (45). This ontology-driven approach to data labeling facilitates data harmonization and enhances interoperability, allowing data conversion between different data models (34).

#### FHIR Extension

Another recommendation is the development of an FHIR extension that can handle nuclear medicine imaging and theranostics data exchange. FHIR has become a powerful standard for exchanging health care data, making it easier for health care systems, software applications, and research platforms to structure and exchange data. A FHIR extension optimized for nuclear medicine would help streamline efforts to collect imaging and theranostics datasets from clinical databases. Similar to other FHIR extensions, the extension could be shared easily across institutions, facilitating efforts to aggregate multiinstitutional datasets.

This effort would require a multidisciplinary working group to generate a comprehensive list of the data elements to be covered by the extension (46). These elements should be aligned with existing FHIR resources, when possible, and all data concepts should be mapped to biomedical ontologies. An FHIR implementation guide would need to be developed to document the extension, including the data elements, resource mappings, and any specific examples of usage. Ultimately, this would be shared with and refined by the wider nuclear medicine community.

#### Formatting Imaging Data

Expectations for the formatting of nuclear medicine imaging data must be established and widely recognized. We recommend that all imaging data be formatted in DICOM. This includes PET, CT, SPECT, and scintigraphic images; projection (raw) SPECT data; AD maps (DICOM Radiotherapy Dose); and contours (DICOM Radiotherapy Structure Set or DICOM-RT). For PET and SPECT list-mode data, no data standard currently exists, which hinders efforts by the research community to share and improve image reconstruction methods. Fortunately, standardization efforts are underway, driven by the Emission Tomography Standardization Initiative (47,48).

An additional challenge is that the DICOM metadata elements that are needed to adequately support quantitative SPECT have not yet been fully standardized. Quantitative SPECT is essential for RPT dosimetry and measuring SUVs. But capturing the information needed to convert the native SPECT images (counts) into quantitative units (becquerels per milliliter) is not straightforward, and each vendor has had to devise its own solution. There are also methodologic metadata, such as how the scanner was quantitatively calibrated, that DICOM does not currently capture. These metadata can be important to the interpretation of and future reuse of imaging data and must also survive the DICOM deidentification process. The nuclear medicine DICOM standard should be updated to keep pace with the latest advancements in quantitative imaging and theranostics.

#### Formatting EMR Data

The integration of nuclear medicine imaging with EMR data is essential for addressing many important research questions. This integration requires connecting imaging data with diverse clinical information sources: laboratory test results (e.g., blood counts), clinical variables (e.g., toxicity scores, disease progression time points), clinical notes (e.g., discharge summaries), and other data types (e.g., multiomics). For meaningful analyses, all of these nonimaging data must be standardized.

Data exchange and interoperability have made significant progress in several areas. The need for data exchange and interoperability has existed nearly since the origins of electronic health care information systems, with HL7 version 2 being released in 1989. This information-exchange protocol is still used in approximately 95% of health care institutions (49). Similarly, the first version of the DICOM standard for exchanging medical imaging data was released in 1985 (50). The spectrum of clinical domains has benefited from the standardization of structured coding systems, terminologies, and systems of standardized data elements. Laboratory test data, for instance, has benefited from the widespread adoption of LOINC as a universal standard for the exchange of structured laboratory test data (22). For nonlaboratory data elements, such as nonimaging diagnostic tests, standardization can often be achieved using other established resources, including the *International Classification of Diseases, 10th Revision*, and SNOMED-CT. FHIR is the most recent health care data-exchange protocol developed by HL7 International. FHIR takes advantage of modern Web technologies and provides granular, structured clinical concepts. Guidance for implementing standards for specific databases or clinical needs can be obtained from profiles from Integrating the Healthcare Enterprise (51).

Some EMR data, however, are unstructured, such as free-form clinical notes, which can prove challenging to analyze systematically across institutions. Ultimately, when integrating EMR data with nuclear medicine data, mapping data elements to recognized terminologies and data standards is recommended, when possible. If this is not possible, comprehensive descriptions of the data in a data dictionary are crucial. Ultimately, these efforts will enable more comprehensive and reliable research.

We also recommend that clinical and research medical databases be designed with the capability to track observations at the tissue, lesion, or region-of-interest level. For example, assigning unique identifiers to specific imaging observations, such as individual lesions, could help link different data types at a granular level. Lesion-level measurements, such as imaging quantitation (PET, CT, MRI), molecular assays (e.g., genomics, proteomics), and AD, could be better tracked and compared. This fine-grained approach to data organization would facilitate more comprehensive data analysis and reporting.

#### Standardized Processes and Documentation

In addition to data standardization, standards should be established to ensure that the processes involved in generating nuclear medicine data consistently achieve high levels of quality and reliability. When laboratory testing became widespread in health care, there were concerns about inaccuracies due to poor quality standards (52). The Clinical Laboratory Improvement Amendment introduced standards for good laboratory measurement practices, requiring rigorous validation and certification processes. Unfortunately, theranostics processes currently lack this rigor. Many institutions have had to quickly develop RPT and dosimetry workflows to meet pressing clinical needs. Although these workflows may be reliable when used correctly by the appropriate personnel, a systematic way of documenting and validating these workflows is needed to ensure their ongoing reliability.

For example, ensuring the traceability of radionuclide activity meters to accredited national metrology institutes, such as the National Institute of Standards and Technology, is crucial for ensuring the consistency of nuclear medicine procedures. Inaccuracies in activity measurements can occur along the entire chain of quantitative imaging and analysis. Yet, standardized processes for certifying the quantitative accuracy of nuclear medicine instruments are lacking outside of clinical trials; there should be an effort to bring those standards into routine clinical practice. Although international audit programs exist to provide guidelines for instrument quality assurance (53), greater quantitative rigor, akin to the Clinical Laboratory Improvement Amendment or clinical trial standards, is needed for routine theranostics.

Moreover, when measurements of nuclear medicine processes are reported or stored in databases, measurement uncertainties also should be calculated and stored. In some instances, these uncertainties might be difficult to directly calculate (e.g., quantitative SPECT accuracy), but documentation of these uncertainties could help improve the comparison, aggregation, and interpretation of quantitative data.

#### Use Case

Building on the example of correlating clinical outcomes with AD measurements in PSMA RPT, data standardization plays a critical role by ensuring that imaging protocols, organ delineation methods, dosimetry software, and parameter settings are consistent and interoperable across institutions. This harmonization allows for meaningful comparisons and data integration, ultimately enabling robust analyses that link RPT dosimetry with patient outcomes. A nuclear medicine data model and FHIR extension optimized for both quantitative imaging and RPT dosimetry would make large-scale collection and exchange of PSMA RPT data significantly easier (54). Moreover, it would ensure that PSMA datasets from different sources have compatible data formats and labeling, overcoming the inherent variability in nomenclature between different data sources. This would facilitate data aggregation and analysis of datasets from different institutions and scanners. It would also ensure that PSMA datasets have comprehensive and standardized metadata, which would help identify compatibility issues or systematic differences between different datasets. This could also help researchers evaluate the impact of different methodologic approaches to PSMA dosimetry. Another benefit of a standardized data model is that it would facilitate the collection and standardization of patient outcome data and toxicity scores, which are crucial to understanding PSMA dose–effect relationships, and could contribute to refining treatment planning and improving patient outcomes. These data could lead to the development or validation of AI tools that perform dosimetry-related tasks or predict patient outcomes.

## DISCUSSION

Nuclear medicine theranostics is poised for tremendous growth, which can have a transformative impact on patient care. New hardware and software technologies, including the development of new theranostics agents (1,2,55–57) as well as AI and large language models, are continuously driving the field forward. However, to fully recognize the potential of nuclear medicine theranostics for optimizing patient outcomes, researchers and developers must be able to pool and exchange datasets, including clinical and research data. This article highlighted the gaps in and opportunities for terminology and data standardization in nuclear medicine. These standards should be systematically developed using existing guidelines for standards development (58,59). Our recommendations are summarized in Table 1.

**TABLE 1.** Overview of Recommendations for Standardization of Terminology, Data, and Metadata

Recommendation, by area	Description
Terminology	
Adopt terminology standards	Journals, databases, societies, and training programs should align their terminologies with those established by biomedical ontologies.
Create an ontology for nuclear medicine imaging and theranostics	Develop NucLex ontology describing all necessary nuclear medicine imaging and theranostics concepts and their relationships
Update existing ontologies	Existing biomedical ontologies, including SNOMED-CT, LOINC, RadLex, NCI Thesaurus, and others, should be updated to better account for unique nuclear medicine concepts.
Monitor new terminology	A group of experts should continuously monitor emerging concepts in nuclear medicine and theranostics and update ontologies as appropriate.
Data and metadata	
Define nuclear medicine imaging and theranostics data elements	Create a comprehensive, standardized data dictionary of data elements that may be collected as part of a research or clinical dataset.
Adopt minimum reporting requirements for theranostics studies	The RPT-TEC group provided a set of variables that should be collected and reported as part of RPT dosimetry studies (31).
Develop a data model for nuclear medicine imaging and theranostics data	Using an existing data model as a foundation, such as OMOP or CDISC, build a data model that organizes and structures nuclear medicine data in a standardized and interoperable way.
Use DICOM for imaging data	Images, contours, and AD maps should be formatted in DICOM.
Develop standardized formats for raw tomographic nuclear medicine data	Vendor-agnostic data formats for list-mode and projection PET and SPECT data are needed to link with corresponding DICOM images.
Develop a nuclear medicine imaging and theranostics FHIR extension	An FHIR extension would support large-scale data queries from scanner systems, EMRs, dosimetry software, PACS systems, and other health care databases.
Update nuclear medicine DICOM class	DICOM needs to better capture quantitative SPECT and dosimetry information.
Capability of lesion-level tracking in clinical and research databases	Instead of the typical examination-level granularity, clinical and research databases should have lesion-level or observation-level granularity to allow for better tracking and fine-grained correlative analysis.
Establish good practice standards for dosimetry	Rigorous standards for RPT measurement practices, similar to those used in laboratory sciences, need to be established.

NCI = National Cancer Institute; RPT-TEC = Radiopharmaceutical therapy–Tisue Effects in the Clinic; PACS = picture archiving and communication system.

Achieving universal adoption of standardized nuclear medicine terminology could present significant challenges. Ontologies, such as NucLex, can offer guidance on preferred terms and synonyms, but reaching a consensus across the field may prove difficult. Existing naming conventions within nuclear medicine may not be entirely consistent or interoperable (60–62), further complicating the standardization process. However, prioritizing standardization over individual preferences is crucial. Even imperfect terms, if used consistently, can effectively represent the underlying concepts.

Although terminology and data standardization are important to achieving FAIR nuclear medicine imaging and theranostics datasets, additional efforts to standardize and harmonize data- generation processes are needed. For example, the heterogeneity of imaging schedules, scan protocols, reconstruction settings, image processing, image evaluation, and dosimetry methods is an obstacle to multiinstitutional data pooling. Recent efforts to analyze global variabilities in quantitative imaging (63,64) and dosimetry (7,8,65) have resulted in an in-depth understanding of sources of variability. Guidelines for quantitative imaging and dosimetry are available (66,67); however, implementation of these in the clinic is lagging. The adoption of these recommendations, in addition to data standardization, could lead to more-consistent datasets that can support multiinstitutional dose–effect studies in RPT and the development of more-generalizable AI models.

## CONCLUSION

The rapid growth of nuclear medicine brings unprecedented opportunities to revolutionize patient care but also introduces significant challenges that must be addressed to sustain progress. To fully harness this potential and drive the field forward, the nuclear medicine community must prioritize the development and adoption of standardized practices. The key gaps and opportunities highlighted emphasize the importance of a unified terminology, robust data standards, and consistent methodologies. Addressing these needs will not only enhance the reliability and interoperability of nuclear medicine data but also accelerate advancements in personalized treatments and broader applications of theranostics.

## DISCLOSURE

Babak Saboury is cofounder of United Theranostics. Carlos Uribe is cofounder of Ascinta Technologies Inc. No other potential conflict of interest relevant to this article was reported.
